# Solitary Langerhans Cell Histiocytosis of the Femur in a Nine-Month-Old Girl

**DOI:** 10.7759/cureus.84389

**Published:** 2025-05-19

**Authors:** Faliq Abdullah, Khaled Alabd, Clare Carpenter

**Affiliations:** 1 Trauma and Orthopaedics, University Hospital of Wales, Cardiff, GBR

**Keywords:** bone lesion, femur, infant, langerhans cell histiocytosis, orthopaedic case, paediatric bone tumour, single-system lch, unifocal lesion

## Abstract

Langerhans cell histiocytosis (LCH) is a rare clonal proliferative disorder of myeloid dendritic cells that can mimic infection or malignancy in paediatric bone lesions. A 9-month-old girl presented with a refusal to weight bear for two days. She was afebrile with mildly elevated inflammatory markers, including C-reactive protein (CRP) of 2 mg/L and white cell count (WCC) of 12×10⁹/L. Radiographs revealed an aggressive lytic lesion in the proximal femur with periosteal reaction. MRI showed a subperiosteal fluid collection suggestive of abscess, bone marrow edema, and adjacent muscle inflammation. The patient underwent curettage of the lesion. Histopathology confirmed LCH, with immunohistochemistry positive for cluster of differentiation (CD)1a, S100, CD68, and Langerin. Molecular analysis detected a *BRAF* V600D mutation. Whole-body MRI confirmed a solitary lesion, consistent with single-system unifocal bone LCH. No systemic therapy was initiated, and the patient was managed conservatively with observation. At six-week follow-up, she demonstrated significant clinical improvement and radiographic healing with no leg length discrepancy. This case highlights the importance of biopsy for diagnosing aggressive bone lesions in infants, as unifocal LCH can mimic osteomyelitis or malignancy. Early recognition and conservative treatment often result in an excellent outcome.

## Introduction

Langerhans cell histiocytosis (LCH) is a rare clonal disorder of dendritic cells characterized by the proliferation of cluster of differentiation (CD)1a-positive, Langerin (CD207)-positive histiocytes that resemble epidermal Langerhans cells [1]. It is the most common pediatric histiocytic disorder, with an incidence of four to eight per million children [1]. Although LCH can present at any age, it is primarily a disease of childhood, with a median age of three to four years [1], and has been reported even in infants under one year old. The disease spectrum ranges from isolated, self-limiting lesions to life-threatening multisystem involvement [1].

The skeleton is the most commonly affected site in pediatric LCH, with bone lesions occurring in up to 80% of cases [1]. A solitary bone lesion, historically referred to as eosinophilic granuloma, represents single-system LCH and accounts for a large proportion of pediatric presentations [1]. Frequently involved bones include the skull, pelvis, ribs, and long bones such as the femur [2]. These lesions are often lytic with possible periosteal reaction, and can resemble osteomyelitis or primary malignant bone tumors on imaging [2]. We present this case from an orthopedic perspective, as our patient’s proximal femur lesion posed diagnostic and management challenges involving both infection and oncology differentials.

## Case presentation

Initial presentation

A nine-month-old girl, previously healthy, was brought to the hospital by her parents due to refusal to bear weight on her left leg for two days. There was no reported history of trauma. The infant was irritable when attempting to crawl or stand, suggesting discomfort in the left hip region. She was afebrile, and systemic review was unremarkable (no rash or organomegaly). On examination, the child appeared generally well. The left thigh and hip showed no swelling or erythema, but she was irritable with passive movement of the left hip. The distal neurovascular status of the limb was intact. There was no known family history of bone disease or similar symptoms, and no genetic testing had been performed on the parents.

**Figure 1:**
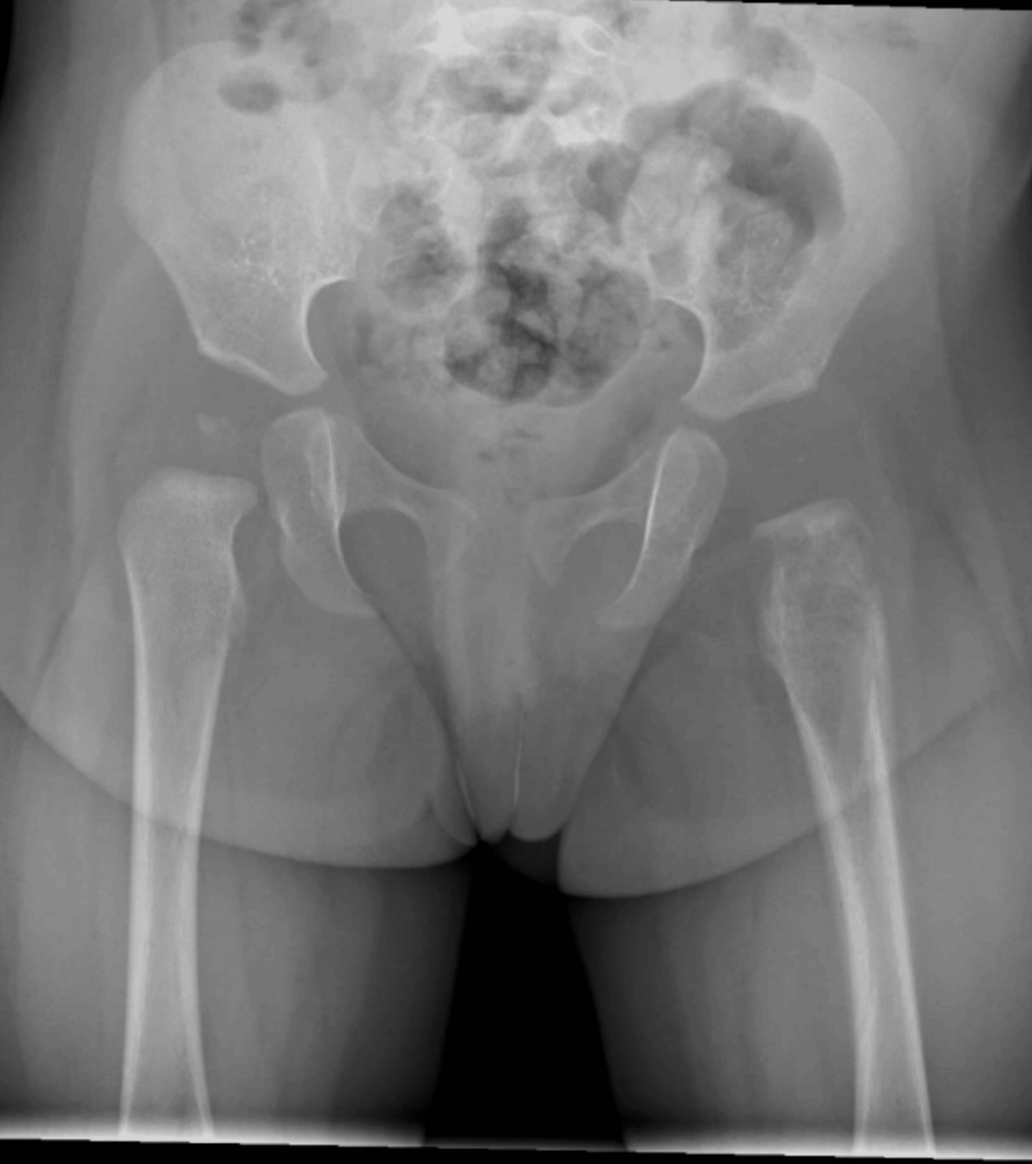
X-ray of the pelvis including the midshaft of both femurs X-ray showing an osteolytic lesion in the proximal metaphysis of the left femur with indistinct margins, cortical destruction, and a layered periosteal reaction along the femoral shaft, suggestive of an aggressive bone process.

Initial laboratory investigations showed a white blood cell (WBC) count of 12×10⁹/L (reference range: 5-15×10⁹/L) and a C-reactive protein (CRP) level of 2 mg/L (reference range: <5 mg/L). These values were within normal limits and did not strongly support a diagnosis of acute osteomyelitis. Nevertheless, due to her inability to bear weight, an urgent X-ray of the pelvis and left femur was obtained. The X-ray demonstrated an osteolytic lesion in the proximal metaphysis of the left femur, characterized by indistinct margins, cortical destruction, and a layered periosteal reaction along the femoral shaft, features concerning for an aggressive bone process (Figure 1).

**Figure 2:**
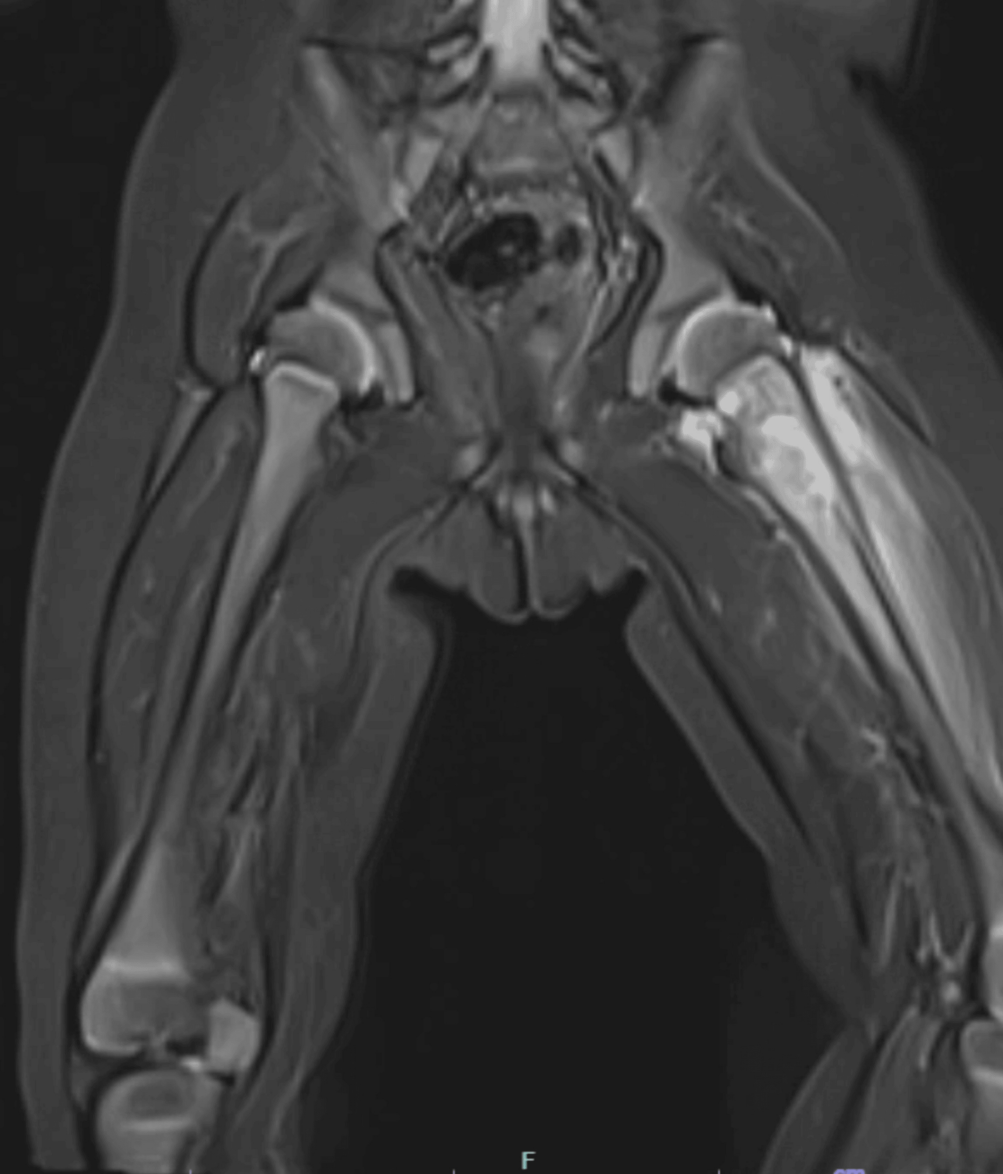
MRI of the pelvis including the midshaft of both femurs MRI of the left femur showing an ill-defined lesion in the proximal metaphysis with bone marrow edema, a subperiosteal fluid collection consistent with abscess, and extensive edema in the adjacent thigh muscles. No discrete soft tissue mass was observed apart from the fluid collection.

MRI of the left femur revealed an ill-defined lesion in the proximal metaphysis, with surrounding bone marrow edema. A subperiosteal fluid collection consistent with abscess was noted, along with extensive edema in the adjacent thigh muscles. No discrete soft tissue mass was identified aside from the fluid collection (Figure 2). Based on these findings, the working diagnosis was a localized bone infection.

The patient was taken to the operating room for an excisional biopsy and curettage under general anesthesia. Intraoperatively, necrotic material was curetted from the metaphysis, and pus-like fluid was drained from beneath the periosteum. Specimens were sent for histopathological analysis and microbial cultures. Empiric intravenous flucloxacillin was initiated postoperatively in view of the suspected abscess.

Investigations

**Figure 3:**
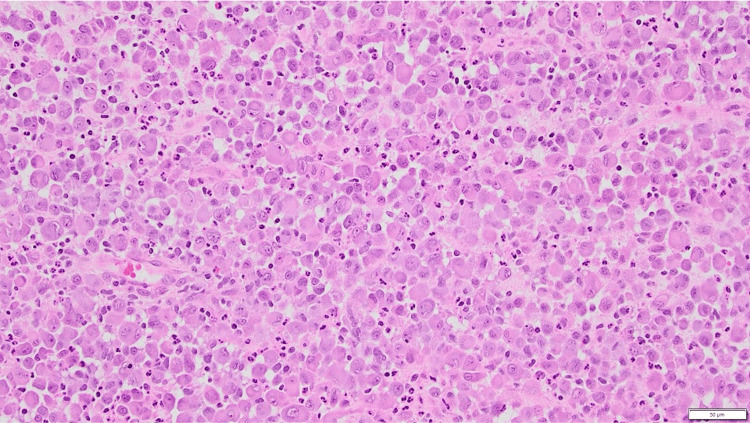
Hematoxylin and eosin stain (H&E) stain Tumour composed of large histiocytoid cells with scattered interspersed neutrophils and eosinophils, X200.

Microscopic examination of the biopsy revealed one fragment of bone with normal trabeculae and fibrovascular marrow. Scattered inflammatory cells, including histiocytes with similar features to those in the soft tissue fragments, were noted (Figure 3). The remaining fragments showed a dense infiltrate of histiocytoid cells, some multinucleated and forming giant cells with osteoclast-like or foreign body-type morphology. Classical Langerhans-type or Touton giant cells were not observed.

The background consisted of a mixed inflammatory infiltrate with neutrophils, eosinophils, and occasional lymphocytes. Areas of necrosis were present, especially where inflammation was most intense, with a predominance of neutrophils and eosinophils. The histiocytoid cells exhibited mild to moderate nuclear pleomorphism without frank anaplasia. Mitotic figures were seen, including at least one atypical mitosis. Occasional emperipolesis and scattered foci of pigment deposition and hemorrhage were noted.

**Figure 4:**
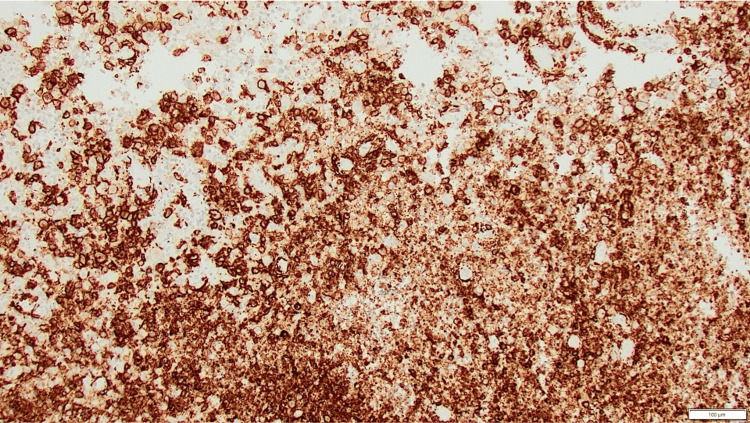
CD1a immunohistochemistry Most cells show cluster of differentiation (CD)1a positivity, X100.

**Figure 5:**
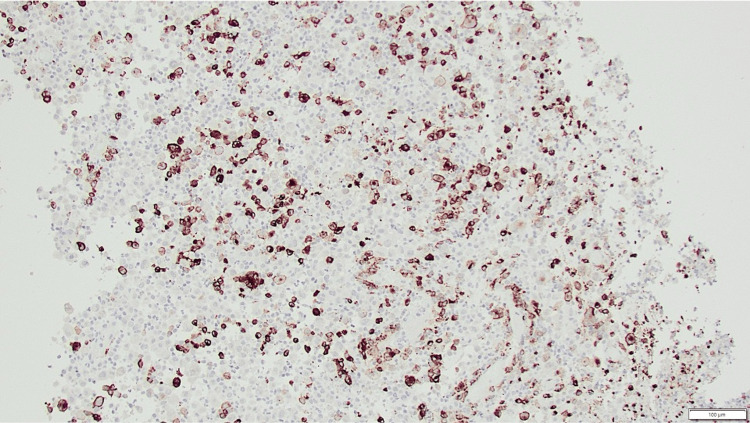
Langerin immunohistochemistry A significant subset of cells show langerin positivity (the negative cells are likely to represent interspersed non-neoplastic (background) histiocytes), X100

Immunohistochemistry supported the diagnosis of Langerhans cell histiocytosis. The majority of lesional cells stained strongly for CD1a (Figure 4) and Langerin (Figure 5). Many cells also expressed CD68 and S100 (not shown). Overall, the features were consistent with a diagnosis of Langerhans cell histiocytosis [3].

The Ki-67 proliferation index was estimated at 10-20%, mostly within the inflammatory infiltrate rather than the lesional population. INI-1 showed preserved nuclear expression. Molecular testing for the *BRAF* V600E mutation was performed due to the known association with LCH. Although V600E was negative, the less common *BRAF* V600D mutation was detected. This rare variant further supported the clonal, neoplastic nature of the lesion [4]. Genetic analysis was performed on formalin-fixed, paraffin-embedded (FFPE) tissue using targeted next-generation sequencing (NGS), which identified a somatic *BRAF* V600D mutation. This variant is classified as pathogenic based on reports in the COSMIC and ClinVar databases, and has been previously described in association with histiocytic disorders [4].

A whole-body MRI (including the spine, pelvis, and long bones) was performed for staging and showed no additional skeletal or soft tissue lesions. There was no evidence of organ involvement; no liver, spleen, or lung lesions were seen clinically or on imaging. These findings were consistent with single-system, unifocal bone LCH. The final diagnosis was Langerhans cell histiocytosis of the left proximal femur, unifocal (single bone), *BRAF* V600D-positive.

Differential diagnosis

The initial differential diagnosis for an aggressive femoral lesion in an infant included both infectious and neoplastic processes. Subacute osteomyelitis (Brodie’s abscess) was strongly considered due to the subperiosteal fluid seen on MRI and the child’s refusal to bear weight. However, the mildly elevated inflammatory markers and absence of fever were atypical for a significant infection. Langerhans cell histiocytosis (LCH) of bone can closely mimic osteomyelitis on imaging [2], as it did in this case, highlighting the importance of biopsy for definitive diagnosis.

Primary malignant bone tumors were another concern. Ewing sarcoma can present in infancy and often affects long bones with a periosteal reaction; however, Ewing sarcoma is exceedingly rare in a 9-month-old infant [5]. Additionally, the absence of systemic symptoms and the MRI findings, specifically the lack of a solid soft-tissue mass, further reduced the likelihood of malignancy. Osteosarcoma was also considered less likely, given the purely lytic nature of the lesion; osteosarcoma in infants is extremely rare and typically exhibits osteoid matrix formation [6].

Alternative histiocytic disorders, such as Erdheim-Chester disease, and benign entities like simple bone cysts, were also part of the differential. However, the patient’s age and the radiographic appearance were most consistent with LCH (historically known as eosinophilic granuloma in its solitary form). In summary, the primary differentials were osteomyelitis versus LCH, with malignancy considered but less favored. This case reinforces that biopsy is essential for evaluating any indeterminate pediatric bone lesion, as clinical presentation and imaging alone may be misleading [2].

The patient was managed as a single-system (bone-only) LCH case. Following surgical curettage and biopsy, no additional local treatment was required. The bulk of the lesion was removed during curettage, and the remaining bone defect was allowed to heal naturally. In accordance with current guidelines, solitary bone LCH can be treated conservatively with curettage and observation, with or without intralesional steroid injection [7]. Complete surgical excision or wide resection is generally unnecessary, as it provides no added benefit and may increase complication risk [7]. In this case, intralesional steroid was not used, although it remains an option in some centers to expedite healing.

Empiric antibiotics initially prescribed for presumed osteomyelitis were discontinued after negative cultures and histologic confirmation of LCH. The family was extensively counseled about the diagnosis and the rationale for an observational approach, that is, LCH is a benign but clonal disorder with a strong tendency to heal. They were advised that chemotherapy or systemic therapy was not necessary, as the disease was confined to a single bone with no evidence of risk-organ involvement. Systemic therapy, such as vinblastine and corticosteroids, is typically reserved for patients with multifocal or multisystem LCH, or for solitary lesions in critical anatomical locations (e.g., vertebral bodies at risk of collapse or skull lesions with intracranial extension) [7,8]. As the femoral lesion had been adequately treated with curettage and showed no features warranting further intervention, the multidisciplinary team, including orthopedic surgery, pediatric oncology, and radiology, agreed on a plan of surveillance. The patient recovered well postoperatively without complications and was discharged home with scheduled follow-up.

Outcome and follow-up

**Figure 6:**
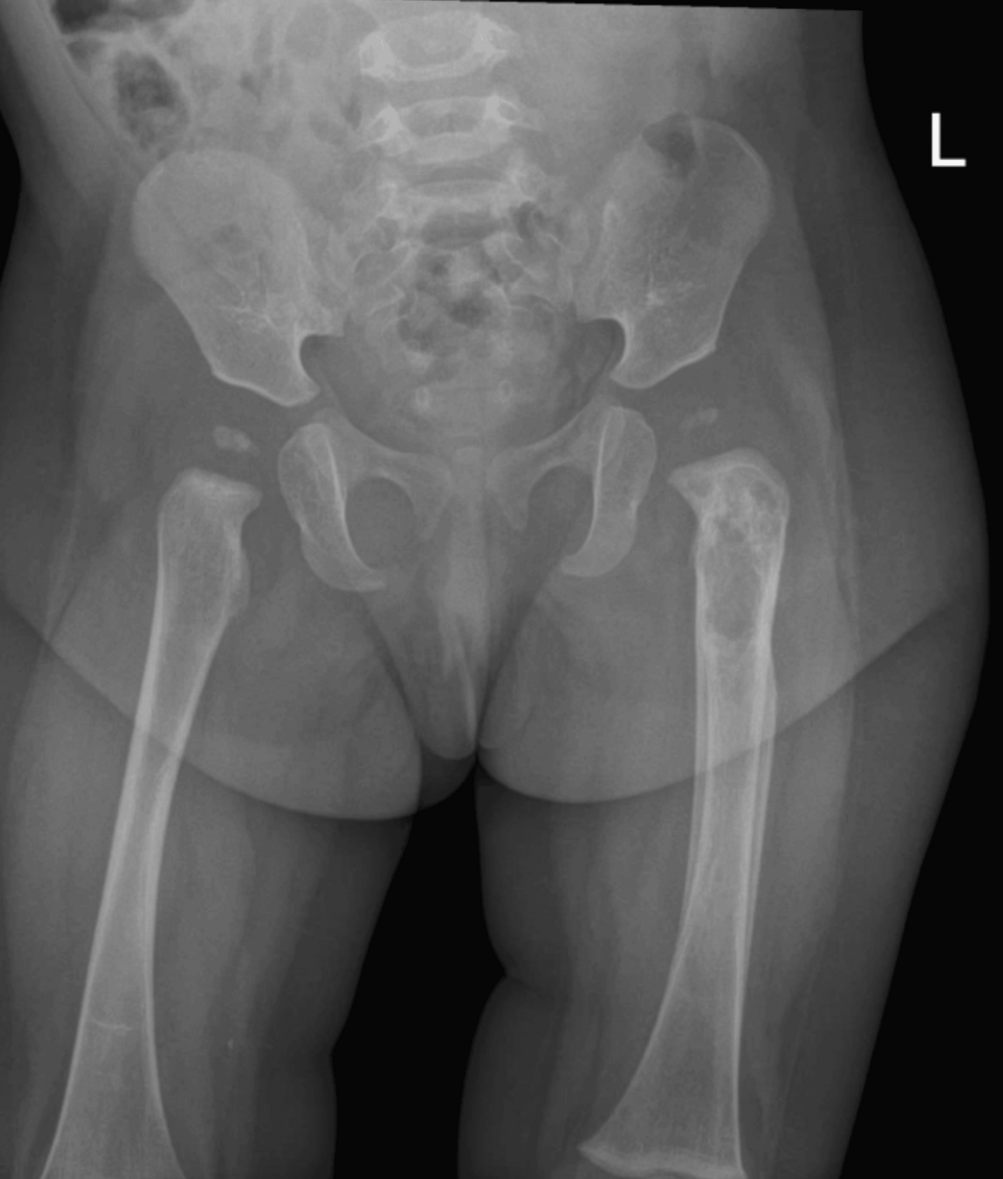
Follow-up X-ray of the pelvis including the midshaft of both femurs Follow-up X-ray of the left femur at six weeks showing new periosteal bone formation and sclerosis at the lesion site, indicating consolidation and early healing of the Langerhans cell histiocytosis lesion.

At six-week follow-up, the infant’s clinical condition had improved significantly. She was comfortable and had resumed crawling and standing with support, now bearing weight on the left leg without distress. Physical examination revealed a full range of motion in the left hip. There were no signs of local recurrence, such as tenderness or swelling. Importantly, limb growth appeared symmetrical, and there was no leg length discrepancy or deformity at this early stage. A repeat X-ray of the left femur at six weeks demonstrated evidence of bone healing. New periosteal bone formation and sclerosis were seen at the lesion site, indicating consolidation (Figure 6). These radiographic findings corresponded with early healing of the LCH lesion.

We plan to continue close observation. The next follow-up will include another X-ray in six to eight weeks to monitor continued bone remodeling. An MRI is also scheduled at three months postoperatively to assess for residual disease and to screen for any new lesions. Thus far, the single-system LCH appears to be healing spontaneously. The expectation is that, over the coming months, the femoral defect will completely ossify and remodel, as has been reported in other conservatively managed cases [9]. We will also monitor the patient’s limb growth over time, as lesions in the proximal femur during infancy could potentially impact the physis. At the most recent visit, there were no signs of growth disturbance.

At six-week follow-up, the patient showed clinical improvement with no signs of recurrence or growth disturbance. Radiographs demonstrated early signs of consolidation at the lesion site. Solitary bone LCH in children is widely reported to have a favorable prognosis, with near-complete survival rates [1] and low recurrence rates, estimated at <20% over five years in unifocal cases [3]. If healing continues as anticipated, no further intervention is expected beyond routine clinical monitoring.

## Discussion

This case illustrates an uncommon diagnosis in an infant with a bone lesion and highlights key considerations in the evaluation and management of Langerhans cell histiocytosis (LCH). Osseous LCH can pose a diagnostic challenge, particularly when presenting with features mimicking subacute osteomyelitis. In our nine-month-old patient, the clinical presentation, refusal to bear weight without fever, and MRI findings, including a subperiosteal fluid collection, initially suggested bone infection. LCH has been reported to mimic osteomyelitis, and abscess-like MRI features, including the “penumbra sign,” can lead to misdiagnosis [2]. For example, one case of tibial LCH in a two-year-old was initially diagnosed as Brodie’s abscess until confirmed by biopsy [2]. Similarly, in our case, imaging alone was insufficient to distinguish infection from a granulomatous lesion. This underscores the importance of early tissue diagnosis. A prompt biopsy allowed for definitive diagnosis, which in turn prevented prolonged antibiotic use and helped avoid unnecessary oncologic intervention.

Although LCH in infancy is uncommon, it is not unprecedented. Historically, multisystem LCH in infants (formerly termed Letterer-Siwe disease) carried a poor prognosis. However, our patient had the most favorable form, single-system, unifocal bone LCH. Modern classification is based on the extent of organ involvement, not historical eponyms. Approximately two-thirds of pediatric LCH cases are single-system, which generally carry an excellent prognosis [1]. Solitary bone lesions (previously called eosinophilic granuloma) may even resolve spontaneously. In our case, staging confirmed a truly unifocal lesion, allowing for conservative management without systemic therapy. In contrast, multisystem involvement, especially when risk organs such as the liver, spleen, or bone marrow are affected, requires chemotherapy and carries a more guarded outlook [1].

A notable finding in this case was the identification of a *BRAF* V600D mutation. Approximately 64% of LCH cases harbor somatic mutations in the Mitogen-Activated Protein Kinase (MAPK) pathway, most commonly *BRAF* V600E [1]. The V600D variant is rare, with few reported cases [4,10]. Its detection in this patient further supports the clonal, neoplastic nature of LCH. Interestingly, such mutations have been identified across a spectrum of histiocytic disorders and may be seen in both aggressive multisystem disease and indolent, spontaneously resolving unifocal lesions [10,11]. In one report, a congenital *BRAF* V600D-mutated lesion regressed without intervention, possibly due to oncogene-induced senescence [4]. While this mutation did not influence immediate treatment in our case, its identification may become relevant in the event of future recurrence or systemic progression.

From an orthopedic standpoint, LCH in weight-bearing bones of young children raises concerns about pathological fracture or growth disturbance. The proximal femur is particularly important due to its mechanical load and proximity to the growth plate. In our patient, no fracture occurred, and the lesion showed early healing following curettage. Literature supports that pediatric bone has a remarkable capacity for remodeling, even after deformity, once the LCH is controlled [9]. Conservative management can yield excellent results within weeks to months [9]. By six weeks, the bone was stabilizing, and the child had resumed near-normal function. Long-term follow-up will remain important to monitor for subtle growth disturbances, such as leg length discrepancy, although none have been observed to date.

This case also underscores the value of a multidisciplinary approach. Although the disease was localized, effective care involved orthopedic surgery (for biopsy and structural support), radiology (for diagnosis and monitoring), pathology (for histological diagnosis), and pediatric oncology (for staging and surveillance). Parental education was essential as well. Initially alarmed by the possibility of cancer, the family needed reassurance that LCH is a clonal histiocytic disorder, sometimes described as a low-grade neoplasm or inflammatory tumor, which in this context behaves benignly. Clear communication helped alleviate their anxiety and build trust in the care plan.

In conclusion, unifocal bone LCH should be considered in the differential diagnosis of lytic lesions in infants and young children, even when they mimic infection. Early biopsy is critical for diagnosis. Solitary lesions can often be treated conservatively with curettage and observation, sparing the child from systemic therapy. This case demonstrates the typically favorable outcomes seen in such cases and adds to the growing understanding of the disease’s genetic and clinical spectrum.

## Conclusions

Solitary bone Langerhans cell histiocytosis (LCH) in infants, though rare, should remain a key differential consideration for lytic bone lesions that mimic infection or malignancy. In cases where infection is the leading clinical concern, early surgical intervention may serve both diagnostic and therapeutic purposes. Nonetheless, it is essential to recognize that when malignancy is part of the differential, a formal diagnostic biopsy should ideally precede surgical management to avoid diagnostic uncertainty. In this case, prompt histological confirmation during the initial procedure enabled accurate diagnosis and appropriate conservative treatment. With careful follow-up, solitary LCH lesions in children typically carry an excellent prognosis and can often be managed without systemic therapy.
